# Wave attenuation through forests under extreme conditions

**DOI:** 10.1038/s41598-022-05753-3

**Published:** 2022-02-03

**Authors:** Bregje K. van Wesenbeeck, Guido Wolters, José A. A. Antolínez, Sudarshini A. Kalloe, Bas Hofland, Wiebe P. de Boer, Ceylan Çete, Tjeerd J. Bouma

**Affiliations:** 1 Unit for Marine and Coastal Systems, Deltares, 2600 MH Delft, The Netherlands; 2 Department of Hydraulic Engineering, Delft University of Technology, 2600 GA Delft, The Netherlands; 3 NIOZ Royal Netherlands Institute for Sea Research, Department of Estuarine and Delta Systems, Utrecht University, P.O. Box 140, 4400 AC Yerseke, The Netherlands; 4 Department of Physical Geography, Utrecht University, P.O. Box 80.115, 3508 TC Utrecht, The Netherlands

**Keywords:** Ecology, Natural hazards, Engineering

## Abstract

Worldwide, communities are facing increasing flood risk, due to more frequent and intense hazards and rising exposure through more people living along coastlines and in flood plains. Nature-based Solutions (NbS), such as mangroves, and riparian forests, offer huge potential for adaptation and risk reduction. The capacity of trees and forests to attenuate waves and mitigate storm damages receives massive attention, especially after extreme storm events. However, application of forests in flood mitigation strategies remains limited to date, due to lack of real-scale measurements on the performance under extreme conditions. Experiments executed in a large-scale flume with a willow forest to dissipate waves show that trees are hardly damaged and strongly reduce wave and run-up heights, even when maximum wave heights are up to 2.5 m. It was observed for the first time that the surface area of the tree canopy is most relevant for wave attenuation and that the very flexible leaves limitedly add to effectiveness. Overall, the study shows that forests can play a significant role in reducing wave heights and run-up under extreme conditions. Currently, this potential is hardly used but may offer future benefits in achieving more adaptive levee designs.

## Introduction

Vegetated foreshores, such as marshes and mangroves, are promoted globally for their capacities in reducing impacts of waves, winds and surges^1–6^. Besides along coastlines there is also potential for reducing wave heights and run-up in rivers and lakes by floodplain vegetation and riparian forests^7,8^. Although the capacity of trees to reduce hydrodynamic energy is intuitive and measured under benign conditions in the field on mangroves^9–11^, their effectiveness under more extreme events is not well substantiated with quantitative evidence. Numerical models generally simplify vegetation by representing it as rigid cylinders^12,13^. Laboratory-flume studies with scaled forests result in parameterized bulk drag values, but these are not yet validated for real-scale extreme situations. Hence, varying flexibility and surface area of leaves, branches and stems, result in scale effects and as a consequence calibrated drag coefficients remain inaccurate^14,15^.

Previous field and laboratory-flume measurements on wave attenuation over grassy vegetated foreshores and plants show that energy dissipation depends on incident wave energy, ambient water depth, and the (vertical) structure and flexibility of vegetation^16–20^. Field studies included significant wave heights up to around 0.6 m^18,21^ with extremes up to 1.0 m. Möller et al. carried out flume experiments exposing real flexible grassy vegetation to maximum wave heights of 0.9 m. In contrast, for forests no such large-scale quantitative evidence exists for storm conditions. Current field observations represent relatively mild conditions with significant wave heights in the range of 0.1–0.5 m^11,22,23^. To obtain a quantitative understanding of wave-attenuation capacity of forests under more extreme conditions, we ran real-scale flume tests with various water levels and significant wave heights up to 1.5 m, using both intact and defoliated 15 years old willows (*Salix alba*) trees.

## Results

### Experimental set up

**Figure 1:**
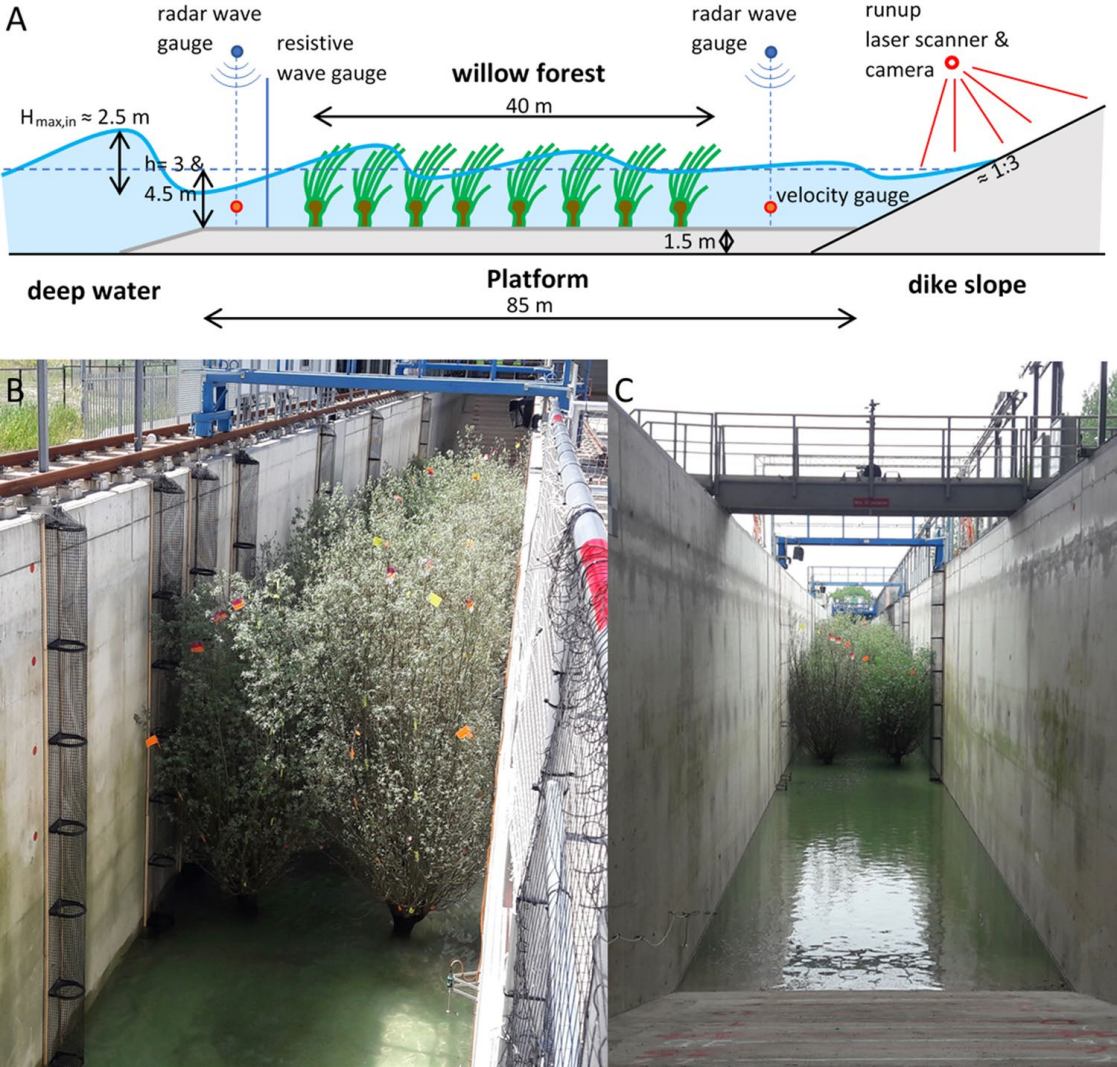
(**A**) Setup of experiments inside the Delta Flume with the most important instruments, (**B**) front top view of willow forest, (**C**) view from the back slope.

We constructed a real-scale willow forest in a wave flume of 300 m long, 5.0 m wide and 9.5 m deep. The forest existed of 32 willow trees that were placed in 16 rows of 2 to build a 40-m long forest on an 85-m-long platform (Fig. 1). The pollard willows (Salix alba) existed of stems that were 15 years old and branches that were 3 years old since the last cutting. Willows were placed with their roots (in a clod) in the sandy base of the platform and fixated by applying a concrete layer of 20 cm as bed. At the back of the forest a concrete levee slope was present (Fig. 1).

Wave attenuation by the willow forest and associated run-up on the slope were measured for different water depths in the forest (h = 3.0 and 4.5 m), significant incoming wave heights at the start of the forest (H_m0, i_ = 0.2 m-1.5 m) and different steepness (S_op_ = 0.02–0.06, where S_op_ = H_m0_/(gT_p_^2^/2π) is the fictitious deep water wave steepness). For willows with leaves only tests with 3.0 m water levels were performed as it was feared that with higher water levels and higher wave heights immediate destruction of the trees would jeopardize further measurements. The tests were designed to limit wave breaking by using water depth ratios of h/H_m0_ larger or equal to 3. The flume is equipped with a reflection compensation system (ARC). All tests were performed with a JONSWAP wave spectrum and a duration of 500 waves per test to allow for a proper statistical analysis of the wave characteristics^24^. Due to the lack of data on specific wave spectra at Dutch willow site locations, a JONSWAP spectrum was chosen because it was considered to best represent the young/growing wave conditions. Test series on willows with leaves, without leaves, with a thinned branch density and, as control, without any willows (bare platform) were executed over a period of three weeks (Table 1, for all tests see Supplementary Information S1). Tests generally lasted for 2–3 days after which the water was lowered. Trees stayed alive and started making new leaves during the tests. Wave characteristics were measured in front of the platform, in front of the forest and behind the forest using resistance wave gauges and radar wave gauges. Wave run-up on the slope was measured using cameras, a laser scanner and visual recordings.

**Table 1:** Summary of the tested hydrodynamic conditions for the different tree forest configurations (series).

Vegetation treatment	Series	H_m0,i_ (m)	H_max_ (m)	T_P_ (s)	h (m)	S_op_ (–)
Willow with leaves and full canopy	2	0.43–0.97	0.74–1.75	2.84–5.57	3.00	0.02–0.05
Willow without leaves and full canopy	3	0.43–1.41	0.72–2.45	2.84–6.85	3.00–4.50	0.02–0.06
Willow without leaves with reduced canopy	4	0.43–1.44	0.78–2.52	2.84–6.85	3.00–4.50	0.02–0.06
No willows	5	0.17–1.43	0.26–2.51	2.84–6.85	0.60–4.50	0.03–0.05

Test series 1 is omitted due to low water depths.

All values are based on the wave height in front of the forest with significant incoming wave height (Hm0,i), maximum wave height (Hmax), wave period (Tp), water depth (h) and wave steepness (Sop). For all tests of these series that are analyzed in this paper see Table S2.

### Reduction in wave height and run-up through the forest

**Figure 2:**
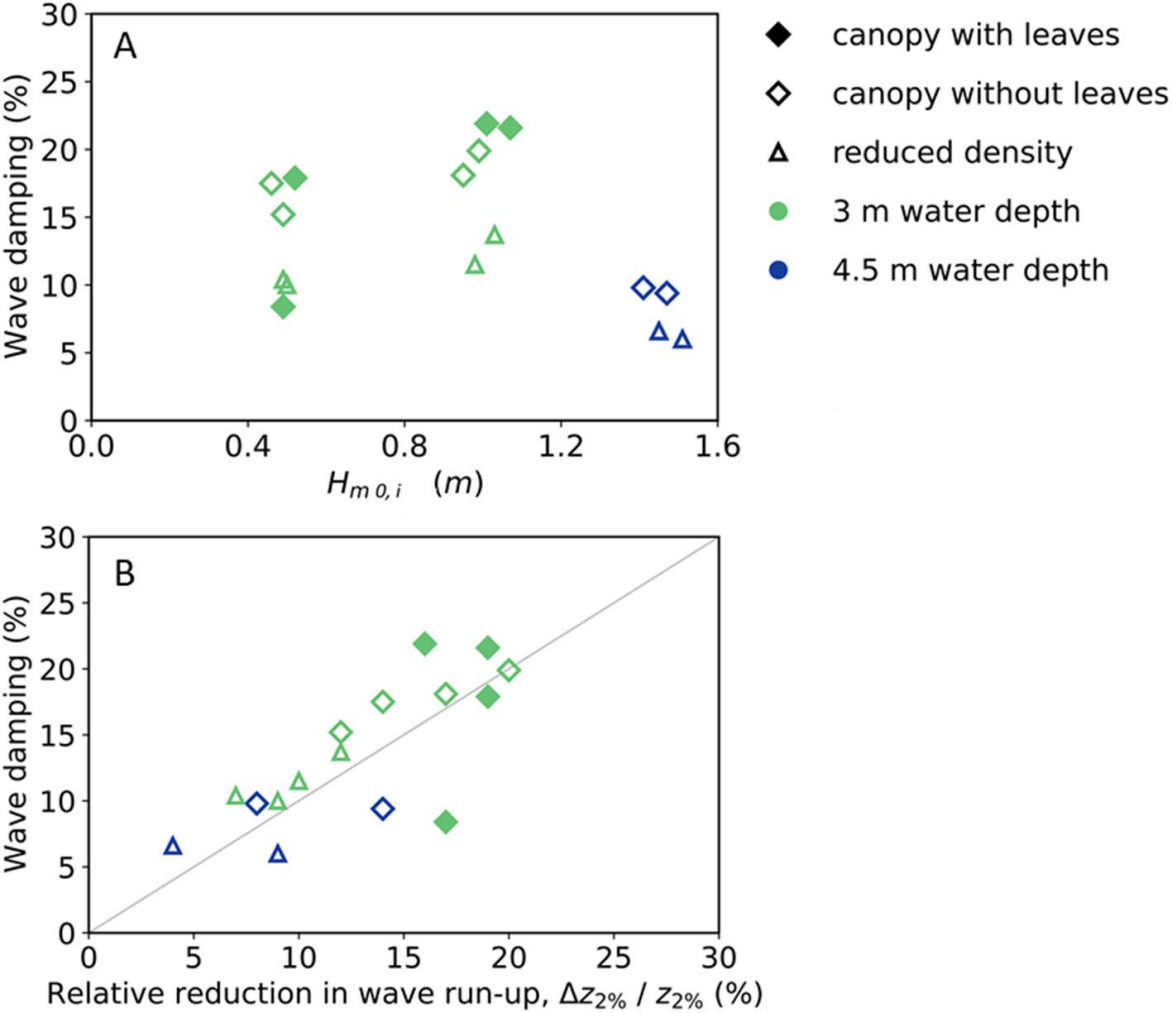
(**A**) Relation between measured wave attenuation (%) and incoming significant wave height (Hm_0, i_). Markers represent the different willow forest configurations (2 = with leaves, 3 = without leaves, 4 = reduced density branches), and the colors show water levels (green = 3 m and blue = 4.5 m), (**B**) Relation between relative reduction in wave run-up on the slope and the wave attenuation through the forest (r = 0.76): line shows the 1:1 line.

The wave attenuation effect of the forest was represented as the measured transmitted wave height behind the willow forest (i.e., with leaves, without leaves, reduced branch density), in reference to the case with bare platform (without willows) (Eq. 1 in “Methods”). Plotting the wave attenuation as function of the significant incoming wave height, H_m0,i_, shows that for constant 3 m water depth and equal tree configuration the wave damping seems to increase somewhat as a function of wave height (Fig. 2A). The maximum wave attenuation by the willow forest is approximately 22% over 40 m. Maximum attenuation is found for the willow forest with leaves and full canopy (Series 2), as could be expected based on the amount of frontal surface areas around the water line. Wave damping with leaves is 1.5–4% (percentage point) higher than for a canopy without leaves (i.e., approximately 20% over 40 m). Wave attenuation with full canopy density but without leaves is 3–7% (percentage point) larger than with a reduced canopy density (i.e., approximately 15% over 40 m). Wave attenuation was found to be strongly dependent on water level. Attenuation for a water depth of 3 m is larger than for 4.5 m. With larger water depths, waves moved through the thinner part of the canopy which also proved more flexible and showed significantly more bending (pers. obs.). Similar effects have been reported with increasing wave heights for salt marshes^25^. As effects of the bottom are already accounted for in our calculation method for wave attenuation, this likely is explained by the fact that the strongest wave damping occurs when the water depth is around the middle of the canopy height (above the trunk), where the tree has most frontal surface area.

The loss of biomass during different test series was relatively small (less than 1% of total branches and leaves biomass). Limited breaking of stems or branches was recorded throughout repeated extreme tests, including average wave heights of 1.5 m and maximum wave heights of 2.5 m. Likely, the extreme flexibility of the willow branches limits the amount of actual breaking but also causes reduction in wave damping with larger wave heights.

The wave attenuation by the willows was also assessed using the measured (reduction in) wave run-up on the slope (Fig. 2B). Plotting the relative reduction in wave run-up height against the relative wave attenuation reveals these two quantities have a similar magnitude of the reduction effect (i.e., up to 20%). However, runup is influenced by both wave height and wave steepness which is represented by the Irribarren number. The exact influence is not clearly defined for Irribarren numbers around 2, such as in these experiments, but with lower wave heights, the Irribarren number increases, which in its turn increases runup again. Hence, it can be expected that the damping of runup is somewhat less than for wave height.

Also, the observed trends are similar, such as an increase in run-up reduction for increasing wave heights and lower run-up reduction for reduced canopy density. Note that in most cases the wave attenuation based on the wave run-up is somewhat lower than the wave attenuation based on the incident wave height. This might be caused by the method used for the separation of incident and reflected waves (MEM) which is based on linear wave theory, see “Methods” section. The test result for a relative reduction in wave run-up of 17% and wave damping of 8% (test T05, filled green square) seems to be an outlier not in line with the rest of the experiments, since the most similar test in Series 3 also produced a wave damping rate of 17% (just as the wave run-up measurement for T05).

### Implications of measurements for wave-vegetation modelling

We utilized the new measurements, to calibrate the spectral wave model SWAN (Simulating Waves Nearshore)^26^. This model was used in similar studies on wave attenuation over vegetated foreshores^8,13,17^ and is frequently used in engineering practice. Suzuki et al. implemented the effects of vegetation in SWAN based on the phase-averaged wave energy dissipation model due to rigid stems for irregular waves^12,27^. The vegetation model is based on bulk wave dissipation (integrated over all wave frequencies), which is dependent on the incoming wave energy, the water depth and the vertical structure of the vegetation (Eq. 2 in “Methods”). A limitation of this vegetation model is that trees are mostly assumed to behave as a rigid material under hydraulic forces^23^. Furthermore, uneven biomass distribution over the vertical and differences between stems, branches and leaves are limitedly included through varying exposed frontal area.

**Figure 3:**
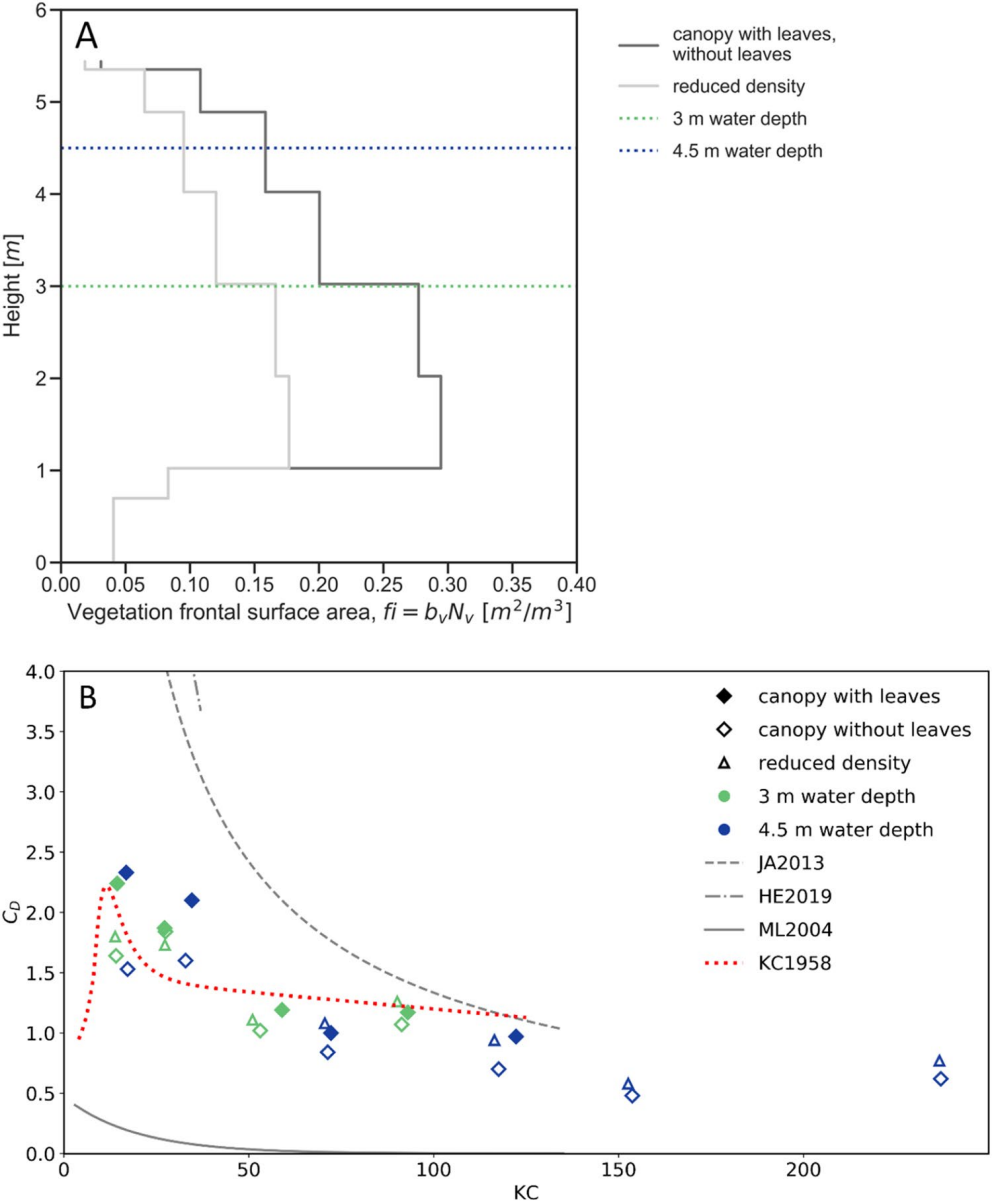
(**A**) Frontal surface area of willow schematization (based in biomass schematization) for test series with full biomass with and without leaves (2 and 3) and with half of the branches removed (4), (**B**) The KC-CD relation for all test series (2 + 3 + 4) and the comparison with the relations by He et al. (2019) (HE2019), Jadhav et al. (2013) (JA2013), Mendez and Losada (2004) (ML2004), Keulegan and Carpenter (1958) (KC1958). For higher KC numbers steady flow would be a straight-line equaling Cd = 1.2.

Generally, vegetation is described by a single branch diameter (b_v_; m) and density (N_v_; m^-2^) per vertical elevation level^13,27^. However, plants have different branches of different sizes and densities. Therefore, here vegetation was represented by a single parameter f_i_(z) (m^2^/m^3^), which described the total frontal area per unit volume, instead of b_v_N_v_ (m/m^2^). This parameter is determined for the present trees by counting all branches at breast level, measuring their diameter and then applying the branching model of Jarvela^28^ (Fig. 3a). Only branches larger than 3 mm were considered. A single representative tree was fully measured to derive a frontal area and determine the frontal surface area distribution over the vertical. This distribution was assumed to hold for all the trees in the flume.

For vegetation-wave models, especially the value of the bulk drag coefficient (${\tilde{C }}_{D}$) has been subject to debate. For flexible vegetation the value of this factor is reduced compared to the value for rigid cylinders because flexible vegetation moves with the flow, which results in less drag force experienced by the vegetation^29^. The ${\tilde{C }}_{D}$ parameter relies on complex physics (e.g., skin friction, pressure differences, swaying of vegetation), which in turn depend on the vegetation properties in relation to the hydraulic conditions^17^. Therefore, instead of determining the ${\tilde{C }}_{D}$ values a-priori, several studies have attempted to calibrate the ${\tilde{C }}_{D}$ values to measurements and relate them to the Reynolds number^30–33^ or the Keulegan-Carpenter number KC^27,34–36^.

Here, the ${\tilde{C }}_{D}$ versus the KC number, ${\widehat{u}}_{s} {T}_{m}/d$ was obtained through optimization of Cd for the present tests by comparing model results and measurement data of the experiments (Fig. 3b and S5). The KC number that is used here is based on the spatial weighted average of branch diameter and velocity, and the orbital motion based on H_s_ and wave period T_p_. Values for low KC numbers are close to the relation by Keulegan and Carpenter^37^ for a single rigid cylinder, as at these KC numbers branches do not bend much. For larger KC numbers the drag coefficient is decreasing, which might be due to increasing motion of the branches that reduces the relative flow velocity. With our measurements we extended Cd values for larger KC numbers, showing that Cd values for larger KC numbers are rather constant. Also, Cd values for large KC number from our experiments are considerably lower than values from previous small-scale experiments^38^. He et al. based Cd on measured wave height decay with models of mangrove trees of up to 35 cm height that included roots, trunk and canopy. These larger values for Cd might be due to wall friction that is incorporated in the Cd values derived from small-scale experiments, the influence of viscous stresses becoming relatively more important at small scale, or to swaying of tree branches which was observed in our experiments. Jadhav et al. based their large values of Cd on wave pressure decay in field measurements on salt marsh vegetation. Mendez and Losada (2004) show low Cd for low KC numbers as these were small scale experiments with very flexible vegetation. For rigid mangrove mimics, Maza et al.^14^ did measure similar values of ${\tilde{C }}_{D}$ between 0.5 and 1.5 at scale 1:6. However, such relations did not exist yet for woody vegetation with a complex vertical structure under extreme conditions on realistic scales (i.e., a wide range of Re and KC numbers).

## Discussion

The present real-scale tests added crucial measurements on the wave attenuation and run-up reduction by forests under extreme conditions and clearly illustrate that floodplain and mangrove forests can contribute significantly to flood safety. This constitutes yet another step towards large-scale implementation of vegetation and levee combinations, or so-called hybrid solutions^39^. These solutions have already been promoted as the way forward under climate change and rising sea levels, as they are considered more adaptive and resilient to uncertainty in environmental boundary conditions^8,40^. However, integrated designs for levees or seawalls in concert with mangroves, marshes and floodplain forests are not yet captured in engineering handbooks and guidelines. Current experiments revealed that areas with only small widths of woody vegetation reduce wave impact and run-up considerably (up to 20%).

Results also showed that the amount of reduction is largely dependent on incoming wave heights and lengths, on present surface area of the vegetation and on movement of branches. Especially, representation of complex vegetation by surface area remains a large unknown in these numerical models and is mostly oversimplified by using cylinder shapes for vegetation representation. Looking in Fig. 2A, there are clear effects of reduced density on wave attenuation. If the plant frontal area is determined correctly, then for high KC and Re numbers, the fitted Cd should become a constant value, approaching 1.2, which is representative for cylinders in uniform flow^41^. This is also illustrated by measurements for rigid mangrove mimics by Maza et al. For our experiments, Cd values with high KC numbers are somewhat lower, which is likely due to flexibility of branches under high wave conditions. Even though KC and Reynolds are defined differently in different studies, which makes comparison between studies difficult, based on this work and previous work we can conclude that there is a region for high KC/RE numbers where Cd approaches uniform flow. These relationships between KC numbers and Cd allow to better calibrate Cd values for situations with high KC numbers, thereby increasing accuracy of future model predictions and avoiding overestimations of the damping effects of trees during storms. More studies with real vegetation and reliable and representative surface areas for field conditions are needed to improve vegetation representation in numerical modelling practices.

Caution should be taken to promote trees as a generic solution for mitigation of extreme hazards as localized studies are always required. With respect to the measurements, the present study deals with emergent vegetation only and the usually observed regime shift with vegetation submergence does not take place and effects of changes in wave profiles or currents are limited (Jacobsen et al., 2019). We also focus on wind waves, as infra-gravity waves are typically not present in situations with riparian vegetation where willow grows. For mangroves growing along more open coasts, infra-gravity waves may play a role. Effects of more diverse forests, such as mangrove forests with different age stands and biomass distribution, have not been explored yet. However, considering the importance of biomass distribution, which was demonstrated by these experiments, more diverse forests may lead to unforeseen results. Although tests with more tree species are obviously desirable, current experiment generated unique first quantitative insights in the wave attenuation capacity of mature trees that can directly be used for modelling and optimizing foreshore management. For application in the field, wave damping is just one of many design aspects that needs to be considered in the safety assessment of a willow forest. Other aspects such as maintenance, uprooting of trees, and damage due to illness or fire are worth exploring. Nevertheless, the first examples of levee foreshore combinations are emerging in the field^42^ and likely more are yet to come.

## Methods

### Wave measurements

The incoming wave height was based on the wave gauge on the platform in front of the forest. This wave gauge was validated for all test series using the radar measurements (RADAC1) in front of the forest. Wave attenuation by the vegetation during each test is defined as the wave height reduction relative to the incoming wave height, which is obtained from the difference between the wave energy spectra measured with and without vegetation:

1 $$\text{Wave attenuation}= \frac{{H}_{m0,\mathrm{ no willows}}-{H}_{m0, \mathrm{willows}}}{{H}_{m0,\mathrm{no willows}}}$$

With: H_m0_ the significant wave height behind the forest.

This method to assess wave attenuation proved most reliable, since it allowed us to exclude effects of wave reflection and damping effects of the platform (which resulted in additional wave attenuation of 2–18%). By using a combination of wave gauge with velocity gauge (EMS) the incident wave height at the foot of the dike (with the exclusion of the reflection component from the dike) could be reliably determined using the Maximum Entropy Method MEM^43^. Note that the distance between the end of the willow forest and the foot of the dike slope was chosen as large as physically possible in the flume (20 m) to limit the effect of evanescent wave modes. Since evanescent wave modes are typically limited to a distance of 0.4 L_p_ from the structure^44^, this was fulfilled by all tests. Also, wave run-up on the slope was measured using cameras, a laser scanner and visual recordings. Wave run-up was obtained through (z_2%,no_willows_—z_2%,willows_) / z_2%,no_willows_).

### Vegetation measurements

Experimental research and field studies on plants (either cultivated or wild), including the collection of plant material, was complied with relevant institutional, national, and international guidelines and legislation. Willows were harvested from private lands where they had been growing for 15 years. They contained stems that were 15 years old and branches that were 3 years old since the last cutting. The branches of the willow trees were categorized into 3 classes based on their diameter at breast height ($DBH$), namely class $1$($DBH>50 mm$) , class $2$ ($20 <DBH \le 50 mm$) and class $3$ ($DBH<20 mm$) (see Fig. S4 in Supplementary Information). Relevant tree data was gathered manually, among which: the total number of branches per class for each tree at breast height, the $DBH$ and branch length for 340 branches, and detailed sketches of 9 primary branches.

### Frontal surface area distribution

The frontal surface area distribution over the vertical was determined for vegetation configurations with and without leaves. The total frontal surface area of the leaves was estimated by the product of the measured total dry weight (38 kg) and a specific leaf area of 145 cm^2^/g^45^, resulting in a value of 1 m^2^/m^3^ for the leaves. However, under wave loads, leaves bend, leading to a more stream-wised position. Assuming that leaves were situated with the smallest frontal area facing the stream, the specific leaf area becomes 1 cm^2^/g (considering a leaf thickness of 0.34 mm, leaf width of 20 mm and dry weight of 0.07 g), which corresponds to a projected surface area of approximately 0.005 m^2^/m^3^. This shows that the contribution of the leaves to the total frontal surface area is limited.

The tapering form of the branches and the occurrence of side branches in the upper layers ($$i>2$$), lead to varying frontal surface areas (equivalent to $${N}_{i}.{b}_{v,i}$$) over the vertical. We used a *branching method* to estimate the total frontal surface area of each tree ($${f}_{total})$$. This method was developed by Jarvela (2004) and originates from the Strahlers ordering scheme. This ordering scheme characterizes *branch orders* (i.e., the conjunction of two branches of order “$$m$$” result in an order “$$m+1$$” branch, starting with the smallest branches, which are assigned to order $$m=1$$). It requires only a few initial tree parameters (such as the average diameter of the smallest branches,$${d}_{min}$$, and the average diameters of the highest order, $${d}_{high}$$, in this case *DBH*) to estimate total frontal area of a tree ($${f}_{total})$$ by using branching factors ($${R}_{B},{R}_{D},{R}_{L}$$) between subsequent branch orders. A more detailed description of the steps is given in the work of Jarvela (2004). A factor of 0.5 was applied to the resulting frontal area per order, to account for the frontal surface area of a cone shaped branch instead of cylinders. Both measurements at breast height and detailed sketches of the primary branches were input to determine the initial parameters and branching factors. These detailed sketches were also used to validate the outcome of this branching methods (See Supplementary Information S1 for further details). With this, the total frontal area for each tree was determined. Although this method is used to predict the total frontal area for trees, its distribution over the height is yet unknown. Therefore, an additional step was added to determine how total frontal area is distributed over height ($$f(z)$$). For this, a single tree was fully measured (i.e., diameters $${b}_{v,i}$$ and the number of branches $${N}_{i}$$ at every meter along the height of the tree). This distribution was assumed to hold for all the trees in the flume.

### Wave dissipation model

The spectral wave model SWAN (*S*imulating *Wa*ves *N*earshore)^26^ following Suzuki et al. was used to model the amount of wave dissipation by willow trees. SWAN was run in its 1D stationary mode, in a Cartesian and regular computational grid. The willow forest was modelled by accounting for 7 vertical layers of vegetation (expressed as frontal surface area), which were assumed to be uniform along the forest length. SWAN is based on the bulk wave dissipation (integrated over all wave frequencies), which depends on the incoming wave energy, relative water depth and vegetation characteristics:

2 $$\langle {\varepsilon }_{v}\rangle =\sum_{i=1:7}\frac{1}{4\sqrt{2\pi }}\rho {\tilde{C }}_{D}{\left(\frac{gk}{2\sigma }\right)}^{3}{f}_{i}\frac{\left({\mathrm{sinh}}^{3} k{\alpha }_{i}h-{\mathrm{sinh}}^{3} k{\alpha }_{i-1}h\right)+3\left(\mathrm{sinh}k{\alpha }_{i}h-\mathrm{sinh}k{\alpha }_{i-1}h\right)}{{3k\mathrm{ cosh}}^{3} kh} {H}_{s}^{3}$$

where $\langle {\varepsilon }_{v}\rangle$ is the averaged wave energy dissipation due to vegetation, ${\tilde{C }}_{D}$ the bulk drag coefficient, $g$ the gravitational acceleration constant, $k$ the mean wave number, $\alpha$ the portion of the water depth covered by vegetation for layer i, $h$ the water depth, ${H}_{s}$ the significant wave height, and f_i_ the total frontal width of vegetation per surface area for layer i, which is equivalent to the generally used b_v,i_N_v,i_.

A correlation was found between the bulk drag coefficient ${(\tilde{C }}_{D})$ and the KC number^37^, as shown in Fig. 3b. The KC number is defined as $KC={\widehat{u}}_{s} {T}_{m}/{D}_{v}$ , where ${\widehat{u}}_{s}$ is the characteristic velocity, ${D}_{v}$ is a representative diameter for the branches of the entire tree, and ${T}_{m}$ is the wave period. The characteristic velocity (${\widehat{u}}_{s})$ is the maximum velocity per layer integrated over the water depth based on linear wave theory. The representative diameter $({D}_{v})$ is determined for each water depth as the branch diameter weighted over the number of branches per vegetation layer and over the layer thickness. The Keulegan—Carpenter number (*KC*) was determined by considering separate layers over the height. This was necessary as willow trees have complex geometries which involve diameter decay and varying branch densities. Several other references use the total width per tree/plant, which has no direct physical meaning in the sense of the original definition of the KC number^37^. Additionally, other studies show differences in flexibility and absence of extra viscous forces that influence results at a small scale.

### Statement on plant materials

Experiments in this research were executed with cultivated willow species (*Salix alba*) of 15 years old that were obtained from a Dutch private site. Trees were replaced with new younger trees. *Salix alba* does not occur on the list of threatened species for the Netherlands and is labeled as stable.

## Supplementary Information

Supplementary Information.
