# Supplementary material for: Wave attenuation through forests under extreme conditions
Source: Sci Rep. 2022 Feb 3;12:1884. doi: 10.1038/s41598-022-05753-3 (PMC8813928; doi:10.1038/s41598-022-05753-3)
Supplement: Supplementary file 1 — Supplementary Information. [file 41598_2022_5753_MOESM1_ESM.docx]

# Supplementary information

### Experimental set-up and measurements


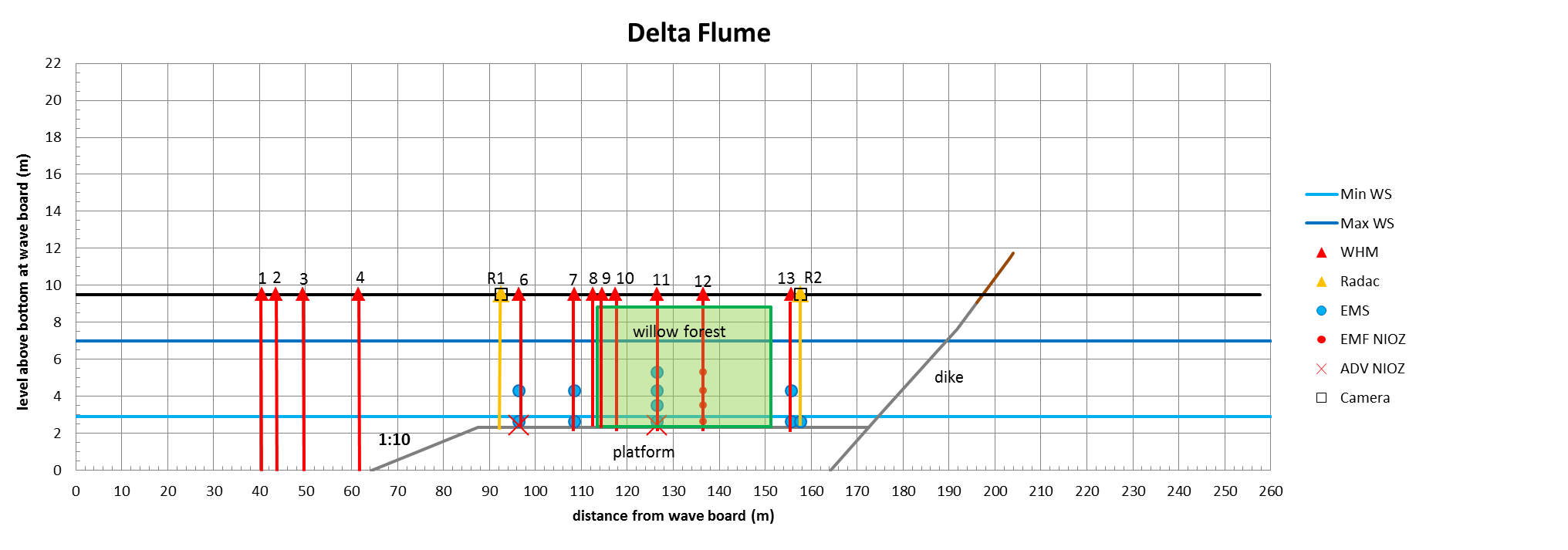


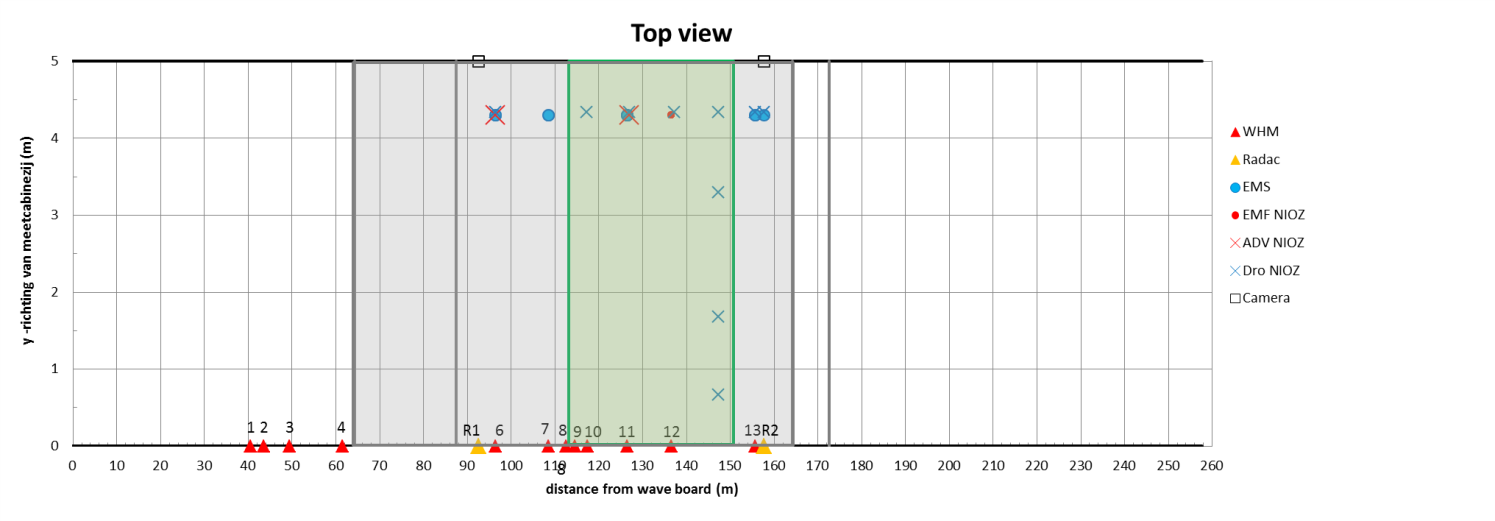


Figure S1. Model setup and instrumentation; Upper panel: Side view, Lower panel: Top view
Abbreviations: WHM = wave gauge (resistance type), Radac = radar wave gauge, DRO = pressure sensor, EMS = electromagnetic velocity sensor, EMF = electromagnetic velocity sensor (provided by NIOZ), ADV = Acoustic Doppler Velocity meter (provided by NIOZ)


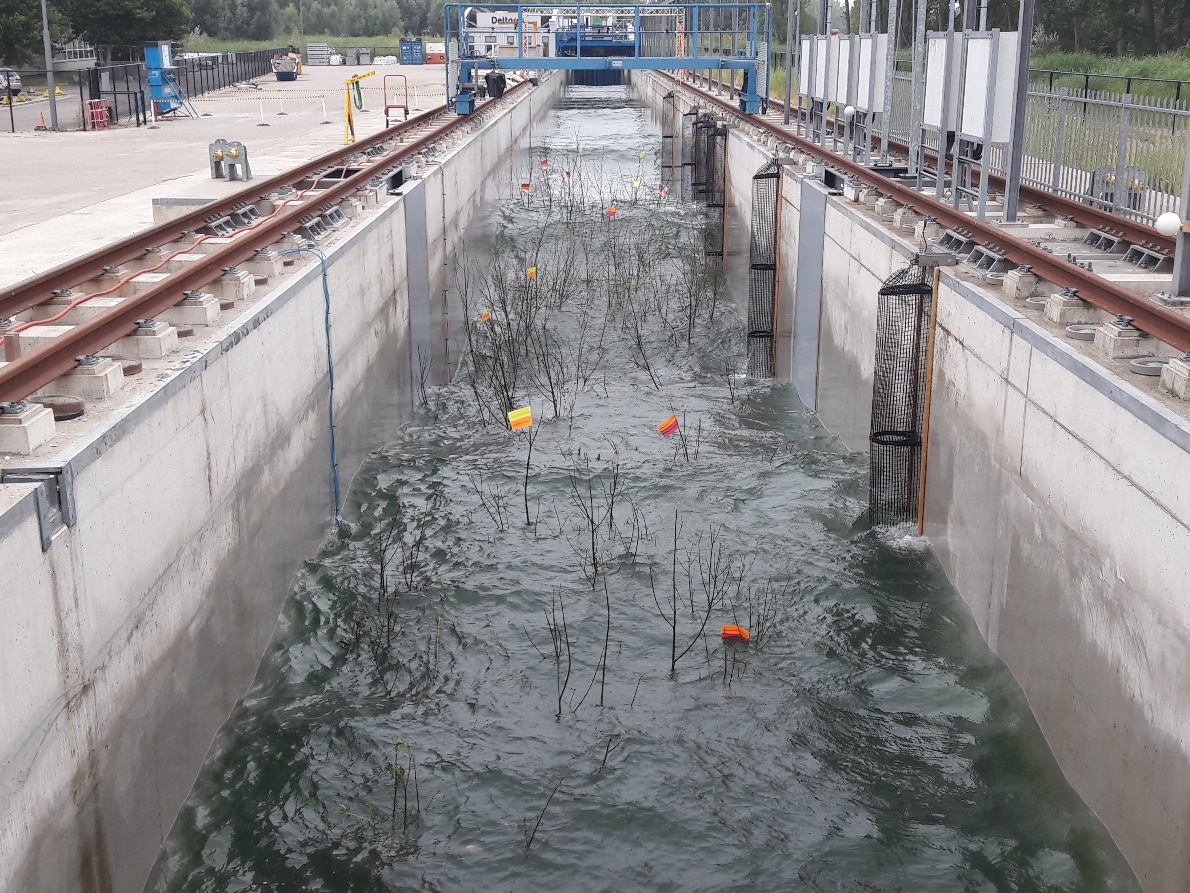


Figure S2. Left: Trees with leaves in flume with 3 m water depth in forest. Right: Trees without leaves with 4.5 meter water level.

Table S2. Hydrodynamic conditions per test measured at the beginning of the foreshore by RADAC1 and wave gauge 6. H_m0,i_ is the significant wave height in front of the forest; H_max_ the maximum wave height in front of the forest; T_p_ the peak period; h the water depth in the willow forest; s_op_ the wave steepness defined as the ratio between H_m0_ and the wave length computed with the mean period of the sea state in deep waters; H_m0,1,willows_ the wave height measured behind the forest at RADAC02 with willow forest; H_m0,1,no willows_ the wave measured behind the forest at RADAC01 without forest; and Cd the bulk drag coefficient as result of SWAN modelling. For the vegetation treatment conditions (series) see Table 1.

| Test | Series | H_m0,i_ [m] | H_max_  [m] | T_P_  [s] | h  Water depth  [m] | s_op_  Wave  steepness  [-] | H_m0,1,willows_  [m] | H_m0,1,_  _no willows_  [m] | Cd  [-] |
| --- | --- | --- | --- | --- | --- | --- | --- | --- | --- |
| 5 | 2 | 0.43 | 0.85 | 2.84 | 3 | 0.04 | 0.37 | 0.41 | 2.24 |
| 6 | 2 | 0.97 | 1.56 | 3.92 | 3 | 0.05 | 0.68 | 0.87 | 1.19 |
| 7 | 2 | 0.47 | 0.79 | 3.93 | 3 | 0.03 | 0.36 | 0.44 | 1.87 |
| 8 | 2 | 0.95 | 1.75 | 5.57 | 3 | 0.03 | 0.75 | 0.96 | 1.17 |
| 13 | 3 | 0.44 | 0.80 | 2.84 | 3 | 0.05 | 0.33 | 0.41 | 1.64 |
| 14 | 3 | 0.95 | 1.64 | 3.57 | 3 | 0.06 | 0.72 | 0.87 | 1.02 |
| 15 | 3 | 0.45 | 0.79 | 3.93 | 3 | 0.03 | 0.37 | 0.44 | 1.84 |
| 16 | 3 | 0.97 | 1.76 | 5.57 | 3 | 0.03 | 0.77 | 0.96 | 1.07 |
| 21 | 3 | 1.40 | 2.19 | 4.80 | 4.5 | 0.05 | 1.26 | 1.39 | 0.48 |
| 22 | 3 | 1.41 | 2.45 | 6.85 | 4.5 | 0.03 | 1.36 | 1.50 | 0.62 |
| 23 | 4 | 0.43 | 0.79 | 2.84 | 3 | 0.05 | 0.36 | 0.41 | 1.8 |
| 24 | 4 | 0.93 | 1.59 | 3.57 | 3 | 0.06 | 0.77 | 0.87 | 1.11 |
| 25 | 4 | 0.43 | 0.78 | 3.93 | 3 | 0.02 | 0.39 | 0.44 | 1.73 |
| 26 | 4 | 0.97 | 1.73 | 5.57 | 3 | 0.03 | 0.83 | 0.96 | 1.26 |
| 29 | 4 | 1.40 | 2.23 | 4.80 | 4.5 | 0.05 | 1.30 | 1.39 | 0.58 |
| 30 | 4 | 1.44 | 2.52 | 6.85 | 4.5 | 0.03 | 1.41 | 1.50 | 0.77 |
| 33 | 5 | 0.44 | 0.76 | 2.84 | 3 | 0.05 | - | 0.41 | 11.9 |
| 34 | 5 | 0.94 | 1.52 | 3.92 | 3 | 0.05 | - | 0.87 | 11.9 |
| 35 | 5 | 0.46 | 0.78 | 3.93 | 3 | 0.03 | - | 0.44 | 11.9 |
| 36 | 5 | 0.96 | 1.67 | 5.57 | 3 | 0.03 | - | 0.96 | 11.9 |
| 37 | 5 | 1.40 | 2.19 | 4.80 | 4.5 | 0.05 | - | 1.39 | 11.9 |
| 38 | 5 | 1.43 | 2.51 | 6.85 | 4.5 | 0.03 | - | 1.50 | 11.9 |


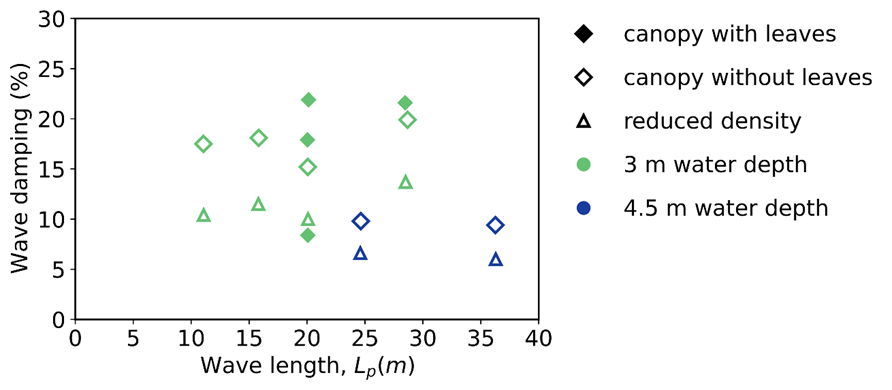


Figure S3. Relation between measured wave damping ([incoming-transmitted]/incoming x 100) and incoming significant wave height at the start of the forest (left panel), or incoming wave length (right panel). Markers represent the different treatments applied in the willow forest and the filling of the markers show tested water levels (3 metres and 4.5 metres).

### Determination of frontal surface area distribution

According to the branching ordering scheme, based on the work of Jarvela (2004), the lowest order begins at the tip of the branches and approaches the highest order (usually the trunk). It requires the following initial parameters, namely: $d_{min},N_{high},d_{high},L_{high}$, where d_min_ is the diameter of the smallest branch, N_high_, d_high_ and L_high_ are respectively the number of branches, the diameter and the length of the highest order branches. A description of the steps can be found in the paper by Jarvela (2004), applied on branches of natural willows.


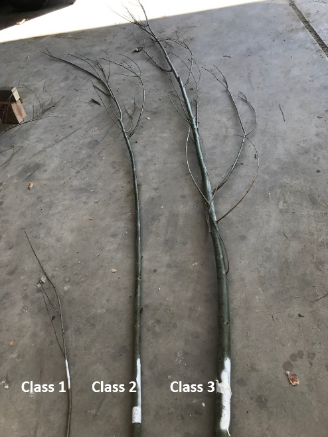


Figure S4. Example of the 3 branch classes

Table S3. Initial parameters for each branch class for one tree.

|  | N_m,high_ (m) | d_high_ (m) | L_high_ (m) | d_min_ (m) |
| --- | --- | --- | --- | --- |
| class 1 (d > 50 mm) | 10 | 0.061 | 3.85 | 0.003 |
| class 2 (20 < d < 50 mm) | 19 | 0.037 | 2.52 | 0.003 |
| class 3 ( d < 20 mm) | 29 | 0.013 | 0.82 | 0.003 |

In this study the values and method were adjusted to account for the branching structure of pollard willows. Firstly, the initial parameters were determined for each branch class as shown in Table S3.

Table S4. Branching factors

|  | ***(m-2)^th^ to (m-1)^th^ order*** | ***(m-1)^th^ to m^th^ order*** |
| --- | --- | --- |
| **R_b_** | 4.19 | 10.56 |
| **R_d_** | 1.71 | 6.26 |
| **R_L_** | 1.44 | 4.50 |

Secondly, instead of averaging the branching factors over all the orders, we maintained separate values for them as shown in Table S4.

Table S5. An example for all the branches of class one for a single tree in the flume

|  | **N (units)** | **d (m)** | **L (m)** | **Frontal area (m^2^)** |
| --- | --- | --- | --- | --- |
| *Branch- order* |  |  |  |  |
| M, primary branch | 10 | 0.061 | 3.85 | 2.35 |
| m-1 | 105 | 0.009 | 0.86 | 0.88 |
| m-2 | 444.2 | 0.005 | 0.6 | 1.55 |
| m-3, smallest branch | 1852.67 | **0.003** | 0.41 | 2.56 |
| Frontal area, total |  |  |  | 7.29 |

The branching factors ($R_{B},R_{D},R_{L}$) were applied starting at the highest order branches (in this case, the primary branches extending from the trunk), to calculate the diameters (d), number of branches (N) and length (L) of the subsequent order branches till the lowest order branch with a diameter of d_min_ =3 mm. The total frontal surface area for each branch class is calculated by N* d* L and taking the sum of the frontal area per order, an example for branches of class 1 is shown in Table S5. By repeating the same procedure for the other branch classes, and taking the sum of these areas, the total frontal surface area of one tree is determined (excluding the frontal area of the trunk). This method assumes linear decay of frontal area over the height and uses cylinder shapes for the branches in each order to calculate the frontal surface area. Therefore, we applied a factor of 0.5 to the frontal area results per order, to account for cone shapes instead of cylinders. Lastly, we used measurements from a single tree to determine the distribution of the total frontal surface area over the height.

### SWAN parameters settings

The following parameter settings were applied in the SWAN numerical model:

- Spectrum: measured
- Breaking: Depth-induced breaking
- Triads: off
- MODE: stationary
- Windgrowth: off
- Quadruple: off
- Wcapping: off
- Refract: off
- friction jonswap constant: 0.07


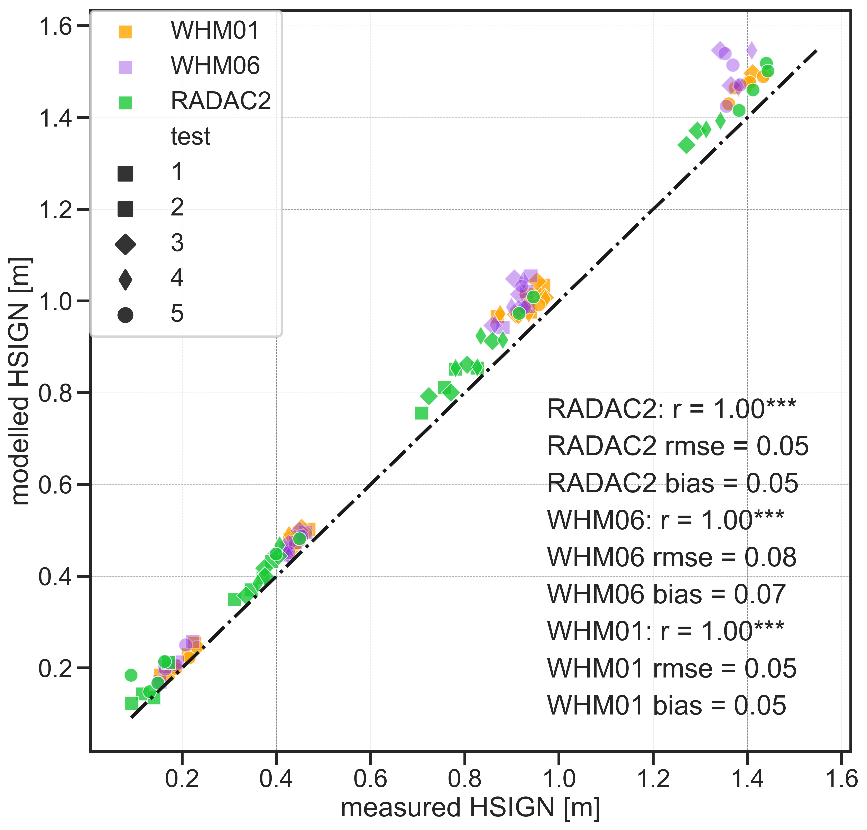


Figure S5. Performance of SWAN model (modelled HSIGN) in comparison to measurements (measured HSIGN). Measurements for two wave gauges in front of the forest (WHM01 and WHM06) and for behind the forest (RADAC2) are shown here.

### Determination of KC

The KC number or period parameter defines the ratio of the distance traversed by a fluid particle during half a wave period to the diameter of the cylinder, according to the definition by Keulegan and Carpenter (1958). The table below shows an overview of the test conditions and ways in determining the KC number, used in some reference studies, indicating possible explanations for deviations seen in Figure 3b. For instance, other references use the total width per tree/plant, which has no direct physical meaning in the sense of the original definition of the KC number.

Table S6. A comparison of Cd-KC relations between different studies.

| **Source** | **Incident hydraulic conditions** | **Vegetation Type** | **Vegetation schematization** | **Determination of Drag coefficient, Cd** | **Determination of KC number** |
| --- | --- | --- | --- | --- | --- |
| Keulegan & Carpenter (1958) | Sinusoidal current | - | Single rigid Cylinder  (The tested diameters ranged from 0.0127 – 0.0762 m) | Morison type equation | $KC={U_{m}T}/D$  Where $u_{m}$ is the maximum intensity of the sinusoidal current, T is the wave period and D is the diameter of the cylinder. |
| Mendez and Losada (2004) | Irregular waves (Flume experiments from Dubi(1995) and Lovas(2000)) | Artificial kelp field  (L. hyperborea kelp) | Rigid cylinders  (D_v_= 0.025 m) | U is the horizontal velocity in the vegetation region. (not relative velocity) | $KC={u_{c}T_{p}}/{D_{v}}$  U_c_ is the maximum horizontal velocity at the **middle of the vegetation field** (x=B/2) and z= -h+αh. Where α is the relative vegetation height. |
| Jadhav et al. (2013) | Irregular waves (Field data) | Salt marsh vegetation (Spartina alterniflora) | Live vegetation  (D_v_= 0.008 m) | Chen and Zhao (2012) formulation.  Where, the **vegetation affected velocity** is used to determine the wave dissipation and Cd. | $KC={u_{rms}T}/{D_{v}}$  U_rms_ is the root-mean-square orbital velocity at **the bed,** considering entire spectrum. |
| He et al. (2019) | Regular waves (Flume experiments) | Mangrove mimics  (Rhizophora) | 1) Stems only  2) Trunk and stems  3) Trunk, stems and Canopy  **(**Dtrunk shape= 8 cm, Dstem= 1 cm, Dcanopy shape= 10 cm (**Not** the individual stems)) | Dalrymple and  Kobayashi formulation | $KC={u_{max}T}/{b_{v}}$  T is the wave period, u_max_ is the maximum horizontal orbital velocity **in front** of the vegetation field **at still water level** from linear wave theory and b_v_ is the vegetation area per unit height of **each plant** normal to wave direction. |

The Length ratio (L=$\frac{l.\omega}{u_{w}}, u_{w}= characteristic velocity;\omega$ = the cyclic frequency; and $l$= branch length) and the Cauchy number (Ca=$\frac{\rho.D.u_{w.}^{2}l^{3}}{EI}, \rho$ = the fluid density; D = diameter of the branch; E= the Youngs’ Modulus; and $I$ = the second moment of inertia ) are shown to be important non-dimensional parameters to describe the motion of flexible stems^46^. According to Jacobsen et al. (2019) , a stem is considered stiff if the Inertia-to-stiffness ratio ($Ca.L/KC)$ < 0.5. For the present experiments, this ratio ranges between 0.1- 45 considering a primary willow branch without side branches, where the lower values correspond to the tests with water depths of 3 m and the higher values to the tests with water depths of 4.5 m. This suggests relatively stiff behavior for the branches during the tests with low water levels and an increase of branch motion during the tests with higher water levels. The branch-density of the primary branches is low (about 14.4 m^-2^), which gives a mean distance between branches of, roughly, 15 times their diameter. This means that the influence of the branches on the $\tilde{C}_{D}$ due to blockage is limited, in the order of 10% ^15^. The Reynolds number for the largest waves based on maximum orbital motion, the significant wave height, and the largest branch diameter class was 10^5^. This means that even though the scale is large, the boundary layer around the branches is still laminar, such that the drag crisis, a fluid dynamics phenomenon which can reduce the drag coefficient and thereby the energy dissipation by a factor of 4, was mostly not reached. The drag on the stem is expected to have reached the drag crisis for a small percentage of the most extreme waves.

Figure S6. Willows in front of a levee in a Dutch floodplain
